# Current Toolset in Predicting Acute Coronary Thrombotic Events: The “Vulnerable Plaque” in a “Vulnerable Patient” Concept

**DOI:** 10.3390/life13030696

**Published:** 2023-03-04

**Authors:** Maria Emfietzoglou, Michail C. Mavrogiannis, Hector M. García-García, Kimon Stamatelopoulos, Ioannis Kanakakis, Michail I. Papafaklis

**Affiliations:** 1 Department of Medicine, University of Ioannina, 45 110 Ioannina, Greece; 2 Division of Cardiovascular Medicine, Radcliffe Department of Medicine, University of Oxford, Oxford OX3 9DU, UK; 3 Section of Interventional Cardiology, MedStar Washington Hospital Center, Washington, DC 20010, USA; 4 Department of Therapeutics, Faculty of Medicine, National and Kapodistrian University of Athens, 157 72 Athens, Greece; 5 Catheterization and Hemodynamic Unit, Alexandra University Hospital, 115 28 Athens, Greece

**Keywords:** coronary disease, atherosclerosis, coronary plaque, myocardial infarction, imaging, shear stress

## Abstract

Despite major advances in pharmacotherapy and interventional procedures, coronary artery disease (CAD) remains a principal cause of morbidity and mortality worldwide. Invasive coronary imaging along with the computation of hemodynamic forces, primarily endothelial shear stress and plaque structural stress, have enabled a comprehensive identification of atherosclerotic plaque components, providing a unique insight into the understanding of plaque vulnerability and progression, which may help guide patient treatment. However, the invasive-only approach to CAD has failed to show high predictive value. Meanwhile, it is becoming increasingly evident that along with the “vulnerable plaque”, the presence of a “vulnerable patient” state is also necessary to precipitate an acute coronary thrombotic event. Non-invasive imaging techniques have also evolved, providing new opportunities for the identification of high-risk plaques, the study of atherosclerosis in asymptomatic individuals, and general population screening. Additionally, risk stratification scores, circulating biomarkers, immunology, and genetics also complete the armamentarium of a broader “vulnerable plaque and patient” concept approach. In the current review article, the invasive and non-invasive modalities used for the detection of high-risk plaques in patients with CAD are summarized and critically appraised. The challenges of the vulnerable plaque concept are also discussed, highlighting the need to shift towards a more interdisciplinary approach that can identify the “vulnerable plaque” in a “vulnerable patient”.

## 1. Introduction

Despite significant advances in the prevention, diagnosis, and management of coronary artery disease (CAD), cardiovascular disease remains one of the leading causes of morbidity and mortality worldwide. CAD accounts for approximately 20% of all deaths in Europe and the United States every year [1], while it is estimated that approximately 20.1 million Americans have CAD. Furthermore, CAD is an underlying cause of death in approximately 1 out of every 5 deaths in the U.S. [1,2].

In the past decades, the quest to recognize high-risk patients (i.e., “vulnerable patients”) as well as “vulnerable plaques” has generated much interest [3]. Fissures and erosions on the intima of coronary arteries were initially described as the origin of thrombosis in the 1960s [4], while the association of myocardial infarction with the rupture or erosion of an atherosclerotic plaque was noted [5,6,7]. The primary cause of coronary plaque ruptures is a lesion called thin-capped fibroatheroma (TCFA). Intracoronary imaging studies in humans using a variety of imaging tools, including intravascular ultrasound (IVUS) and optical coherence tomography (OCT), have proved to be particularly useful in the in vivo identification and visualization of TCFAs and other high-risk plaque characteristics [8]. Invasive imaging showed that other than TCFAs, plaque erosion and calcified nodules may also give rise to thrombotic events [9,10,11]. However, results of studies that have been published lately and have used invasive imaging techniques for “vulnerable plaque” identification have failed to demonstrate high prognostic performance or clinical utility of plaque imaging, thereby indicating that additional information is needed beyond the “vulnerable plaque” concept to identify vulnerable patients [12,13].

Currently, research is shifting towards a holistic approach of identifying the “vulnerable patient” and indicating the “burden” of disease as a major predictor of cardiovascular risk [14]. To this end, risk stratification scores, circulating biomarkers, antibodies, and genetic scores can also provide valuable insight into distinguishing patients at risk of an acute coronary event. Further, assessment of invasive and non-invasive coronary hemodynamic indices has been associated with adverse outcomes, while the use of non-invasive technology, including coronary computed tomography angiography (CCTA), is being proposed as a screening modality to exclude obstructive CAD, even in low-to-moderate risk, asymptomatic individuals, in whom more invasive techniques cannot be performed.

Here, available diagnostic modalities used for identifying high-risk plaques and detecting the progression of atherosclerosis in patients with suspected or diagnosed CAD are reviewed. In addition, contemporary challenges of the “vulnerable plaque”-only concept are discussed, and alternative strategies for identifying the “vulnerable plaque” occurring in a “vulnerable patient” are summarized.

## 2. Pathophysiology of Coronary Atherosclerosis

Plaque morphology goes through various phases before leading to an acute thrombotic event [15,16]. The earliest change is *intimal thickening* and includes layers of smooth muscle cells (SMCs) as well as extracellular matrix without lipid deposits, foam cells (lipid-laden macrophages), and thrombosis. An *intimal xanthoma* or *fatty streak* is a lesion primarily consisting of abundant foam cells interspersed within an SMC- and proteoglycan-rich intima [17,18]. *Pathologic intimal thickening* (PIT) represents the earliest stage of progressive atherosclerosis and is distinguished by the dispersion of surface SMCs and a proteoglycan matrix with lipid accumulation and focal deposits of calcium [19]. Notably, macrophage infiltration of the lipid-rich pool together with the death of these cells results in the conversion of the lesion to a more advanced one called *fibroatheroma* [20]. Fibroatheroma lesions are divided into “early core” and “late core”, depending on the stage of necrosis (early or late) of their cores. Early necrotic cores typically have cholesterol clefts, macrophages, and proteoglycans or collagen, while late cores usually consist of numerous cholesterol clefts and cellular debris with a characteristic absence of extracellular matrix [21,22]. As the fibroatheroma lesion progresses, hypoxia, oxidative stress, and macrophage-induced inflammation can promote the production of vascular endothelial growth factor (VEGF), leading to neovascularization. These neovessels inherently lack SMCs and gap junctions in the endothelium and, thus, are prone to leak, resulting in *intraplaque hemorrhage* (IPH), necrotic core expansion, and, ultimately, plaque rupture [23].

**Figure 1:**
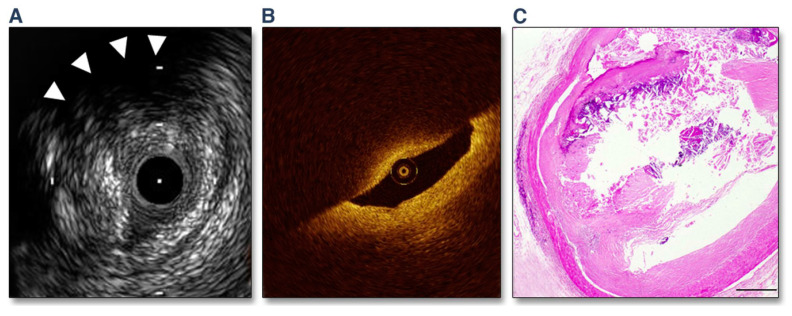
“A Representative Case of OCT- and IVUS-Defined TCFA. (**A**) Grayscale IVUS showing a plaque burden of 82% and a remodeling index of 1.31. Backward signal attenuation behind the plaque and without dense calcium was observed at 10 o’clock (arrowheads). (**B**) A corresponding OCT image indicated signal-poor lesions with an overlying signal-rich band. The minimum fibrous cap thickness was 50 μm. (**C**) A corresponding histological image showing large necrotic cores covered by a thin (50-μm) fibrous cap (hematoxylin-eosin stain, scale bar = 500 μm).” Reprinted from Fujii K, et al. (2015) [24], with permission from Elsevier.

TCFA, the reputed “vulnerable plaque” (i.e., a precursor of plaque rupture), is distinguished by the loss of SMCs, the lipid-rich extracellular tissue, and the abundant inflammatory infiltrates (macrophage predominance). The extent of the lipid core, the fibrous cap thickness, and its structure are indicators of plaque vulnerability; a cap thickness ≤65 μm is considered thin. A representative TCFA with its corresponding IVUS and OCT image is shown in Figure 1 [24].

Although plaque rupture originating from TCFA accounts for most acute coronary syndromes (ACS), ACS can also be generated from plaque erosion or a calcified nodule. As mentioned above, plaques that rupture tend to have a thin fibrous cap, a large lipid core, and abundant macrophages along with a fibrin-rich thrombus. On the contrary, plaque erosion tends to occur in plaques that have little to no lipid core, many proteoglycans and glycosaminoglycans, abundant neutrophils and SMCs, and a platelet-rich thrombi [25]. Additionally, the prevalence of plaque erosion is higher in young females and smokers [26]. According to an in vivo OCT study, individuals with underlying plaque erosion are more likely to have delayed healing after stenting [27]. Finally, complicated *calcified nodules* account for a minority (<10%) of patients with ACS. These are characterized by fibrous cap disruption and thrombi with dense nodules of calcium. They appear to be located predominantly in the mid-right coronary artery and left anterior descending artery, where there is maximal coronary tortuosity. Additionally, they tend to occur in heavily calcified vessels of patients with advanced age or chronic kidney disease [28].

Although it has been well-established that these three distinct pathologies (plaque rupture, plaque erosion, and calcified nodule) can generate ACS, individuals with ACS are most commonly managed with coronary stenting. However, stenting can lead to complications, such as stent thrombosis and restenosis, and thus, certain patients with ACS may benefit from tailored therapy with other interventional treatment modalities, such as isolated thromboaspiration and drug-coated balloons, based on the underlying pathophysiological entity. To this end, several studies have indicated that, for example, ACS caused by plaque erosion could be treated with antiplatelet drugs rather than stent implantation [29,30]. Additionally, in the EROSION study (“Effective Anti-Thrombotic Therapy Without Stenting: Intravascular Optical Coherence Tomography–Based Management in Plaque Erosion”), selected individuals with ACS arising from plaque erosion that were prospectively enrolled and received antiplatelet therapy without stenting had favorable 1-month and 1-year clinical outcomes [31,32]. These results confirmed the merits of a more customized therapy in patients with ACS, but due to the small sample size, further research has to be conducted before this therapeutic paradigm is incorporated into clinical practice.

## 3. Identifying the Vulnerable Plaque

### 3.1. Invasive Imaging in Medical Practice

IVUS is considered the gold standard technique to assess plaque burden, and IVUS virtual histology (IVUS-VH) uses radiofrequency signals to examine anatomic features of the “high-risk plaque”. However, IVUS has relatively low spatial resolution compared to OCT, rendering it unable to measure the thickness of a fibrous cap over a lipid core, as well as to verify plaque erosion. Moreover, IVUS has a relatively low ability to differentiate the amount and distribution of the plaque lipid content.

**Figure 2:**
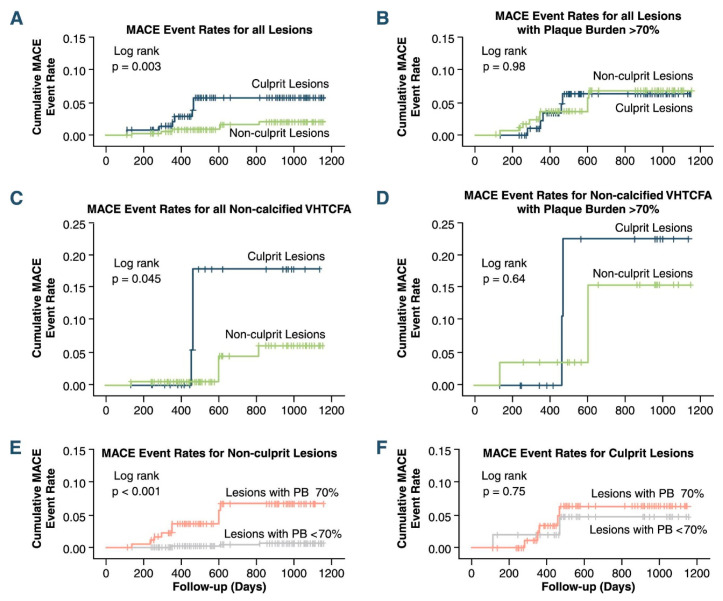
“Kaplan-Meier plot of cumulative MACE Rates from the VIVA (VH-IVUS in Vulnerable Atherosclerosis) study. (**A**) All lesions, (**B**) lesions with plaque burden (PB) 70%, (**C**) all non-calcified virtual histology intravascular ultrasound thin-capped fibroatheroma (VHTCFA), (**D**) non-calcified VHTCFA with PB 70%, (**E**) non-culprit lesions, and (**F**) culprit lesions. MACE: major adverse cardiac event.” Reprinted from Calvert PA, et al. (2011) [33], with permission from Elsevier.

The prognostic value of IVUS-VH in identifying plaque characteristics associated with adverse events was studied in three clinical studies: “Providing Regional Observations to Study Predictors of Events in the Coronary Tree” (PROSPECT), “VH-IVUS in Vulnerable Atherosclerosis” (VIVA), and the “European Collaborative Project on Inflammation and Vascular Wall Remodeling in Atherosclerosis-Intravascular Ultrasound Study” (AtheroRemo-IVUS). The PROSPECT study [13], the VIVA-VH study [33], and the AtheroRemo-IVUS study [34] showed that (1) a minimum lumen area <4 mm^2^, (2) an increased plaque burden (>70%), and (3) TCFA can identify lesions prone to progress and lead to ACS (Figure 2). However, the positive predictive value (PPV) of these three characteristics in identifying culprit lesions was low (18.2%) in the PROSPECT study. Although all three studies indicated that there was an association between high-risk plaque features and MACE, there are significant limitations: (1) in the PROSPECT study, the event rate was low, and the vast majority of events consisted of rehospitalizations for unstable or progressive angina; (2) the VIVA study had a small number of patients that undermines the reliability of the results; and (3) in the AtheroRemo-IVUS study, IVUS-VH was performed only in one vessel, as opposed to all-vessel imaging used in the PROSPECT and VIVA studies, and correlations were made on an individual-level basis, as opposed to the specific-to-lesion manner used in the PROSPECT and VIVA studies.

OCT is broadly used for plaque characterization, especially for the detection of TCFA and rupture [35]. OCT provides unsurpassed spatial resolution (10 times higher than IVUS) and manages to discriminate plaque elements, and also captures macrophage accumulations in the vessel wall [35,36]. Macrophages, in the context of a fibroatheroma, may be depicted by intravascular OCT as regions of increased signal that are more intense than the background noise [37]. Furthermore, OCT is currently the imaging modality providing the highest accuracy for measuring fibrous cap thickness, which can help differentiate among fibroatheromas using 65 microns as the cut-off. Studies with OCT technology have indicated that a larger lesion and increased plaque burden (when visible by OCT) might suggest an increased risk of acute coronary events. OCT can also estimate the therapeutic effect of various agents on plaque characteristics. To illustrate, various studies have used serial OCT to assess the effect of statins on plaque stabilization and fibrous cap thickness (FCT) in patients with ACS [38,39]. Results from a systematic review and meta-analysis of these studies have shown that statins can lead to an increase in FCT with the magnitude of the effect varying with the different statins [40]. Similarly, the randomized, controlled HUYGENS study was conducted to assess the impact of the proprotein convertase subtilisin kexin type-9 (PCSK-9) inhibitor evolocumab on atherosclerosis regression in patients treated with the maximum statin dose (NCT03570697) [41]. The effect was determined using OCT measures of plaque composition, and the results showed that combining statin and evolocumab contributes to plaque stabilization and regression [41,42]. Serial multimodality intracoronary imaging, including OCT, IVUS, and near-infrared spectroscopy (NIRS), have also been used to determine the effects of the PCSK-9 inhibitor alirocumab when added to statin therapy for the treatment of ACS in the PACMAN-AMI double-blind, controlled, randomized trial [43]. The results showed that the addition of alirocumab contributed to atheroma regression in non-culprit arteries [44]. Another important aspect of OCT is that it can identify microchannel networks within plaques that suggest the presence of neoangiogenesis [45]. Neoangiogenesis augments blood flow, and thus increases inflammatory cells and cytokines in the atheromas. However, in contrast to IVUS, one of the major limitations of OCT technology is its limited depth of penetration, and thus imaging the outer vessel wall or estimating plaque burden is rather problematic.

**Figure 3:**
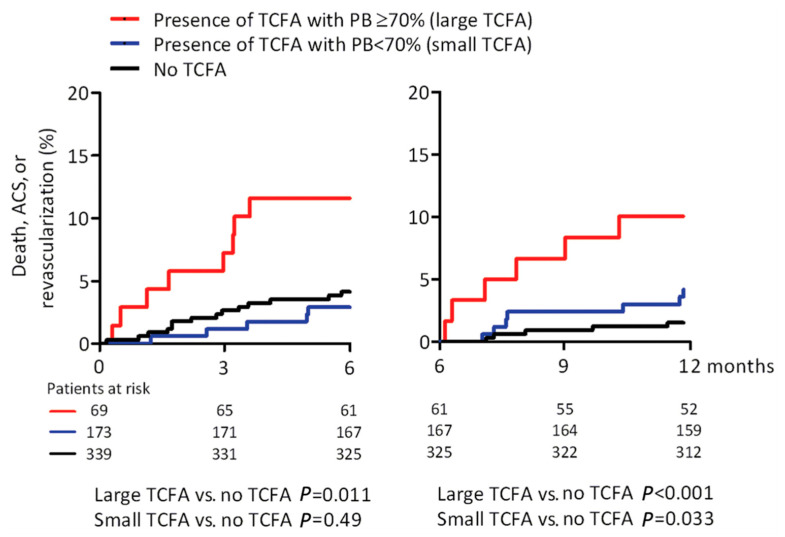
“Associations of short-term and long-term major adverse cardiac events in the ATHEROREMO-IVUS study. *p*-values are obtained with the log-rank test. Overall *p*-value 0–6 months is 0.009; overall *p*-value 6–12 months is 0.002. PB, plaque burden; TCFA, thin-cap fibroatheroma.” Reproduced from Cheng JM, et al. (2014) [34], with permission from Oxford University Press.

NIRS—a tool widely used to discover the composition of substances—has been investigated as a potential technique to ascertain the chemical constituents of coronary plaques [46,47]. NIRS is capable of recognizing lipid components (lipid core plaque—a potential clinical correlate of the “vulnerable plaque”), particularly lipid-rich TCFAs. Madder et al. explored the relationship between large, lipid-rich plaques (LRPs) identified by NIRS technology at non-culprit segments (locations of a culprit vessel that were not stented) and future major adverse coronary and cerebrovascular events (MACCE) [48] (Figure 3). Large LRP was defined as “a maximum lipid core burden index in 4-mm (maxLCBI4mm) ≥500”. MACCE developed in 58.3% of individuals with large LRP as opposed to only 6.4% of patients with a maxLCBI4mm of less than 500. NIRS’s greatest deficiency, though, is its inability to provide information regarding the lumen, plaque anatomy, and morphology. To this end, a hybrid NIRS-IVUS catheter has been developed, providing simultaneous chemical and structural data [49]. Notably, NIRS-IVUS is unique in that it is the only hybrid diagnostic intravascular technology that has been approved for use in clinical practice worldwide [50]. An OCT-NIRS device has also been developed at the Massachusetts General Hospital in the U.S. [51]. This catheter is aimed to provide both OCT and NIRS data with a single pullback, delivering both structural and chemical information for more accurate identification of “vulnerable plaques” and “vulnerable patients”. A commercial OCT-NIRS catheter is currently being developed by SpectraWAVE, Inc., and will soon be available for clinical use [52].

### 3.2. Novel Invasive Imaging Modalities

To date, the value of invasive imaging modalities in predicting acute thrombotic events remains low for clinical utility (Table 1). However, novel invasive modalities are emerging, and aside from NIRS-IVUS technology, an abundance of other hybrid imaging tools have also been introduced lately. The combination of near-infrared fluoroscopy (NIRF) with OCT is a prosperous novel strategy to simultaneously assess molecular and morphological aspects of atheromas in the coronary tree [53]. Molecular imaging is another novel field that intends to capture molecular and biological aspects of organisms by injecting specially designed imaging substances and then using matched imaging modalities. The hybrid catheter (NIRF-OCT) aims to link structural data from OCT with NIRF data, and can visualize in vivo lumen morphology and inflammation when used in animals [54].

Intravascular photoacoustic (IVPA) technology is a diagnostic technique particularly useful for exploring how lipid accumulations are distributed in the coronary arteries [55]. Compared to NIRS, IVPA imaging has increased depth resolution and, thus, it can help identify the specific location and volume of the lipid deposits within the atherosclerotic plaque and its relation to the lumen border. Nevertheless, similar to other novel modalities, there are technical and regulatory limitations that have to be surpassed before IVPA is incorporated into medical practice.

**Table 1:** A summary of studies that have assessed the value of invasive imaging modalities in predicting adverse coronary events.

Modality	Study *	N	Independent Predictor(s)	Endpoint(s)	Mean Follow-Up	Hazard Ratio	*p*-Value
**IVUS & VH**	PROSPECT (“Providing Regional Observations to Study Predictors of Events in the Coronary Tree”) Stone et al. [13]	697	Plaque burden ≥ 70%	Non-culprit MACE	3.4 years	5.03 (2.51–10.11)	<0.001
			Minimal lumen area ≤ 4 mm^2^			3.35 (1.77–6.36)	<0.001
			Thin cap fibroatheromas			3.21 (1.61–6.42)	0.001
	Inaba et al. [56]	697	Negative remodeling index	Non-culprit MACE	3 years	2.39 (1.07–5.34)	0.033
			Positive remodeling index			2.34 (1.00–5.44)	0.049
	Zheng et al. [57]	697	Distance from ostium to max necrotic core site	Plaque rupture	NA	OR 0.86 (0.76–0.98)	0.02
			External elastic membrane area			OR 1.14 (1.11–1.17)	<0.0001
			Plaque burden			OR 2.05 (1.63–2.58)	<0.0001
			Right coronary artery location			OR 2.16 (1.25–3.27)	0.006
			Calcium			OR 0.09 (0.05–0.18)	<0.0001
**Radiofrequency -IVUS**	AtheroRemoIVUS (“The European Collaborative Project on Inflammation and Vascular Wall Remodeling in Atherosclerosis—Intravascular Ultrasound Study”) [58]	581	Minimal lumen area ≤ 4 mm^2^	MACE	4.7 years	1.49 (1.07–2.08)	0.020
			Plaque burden ≥ 70%	Non-culprit MACE		1.66 (1.06–2.58)	0.026
**Angiography & IVUS plus** **CFD**	PREDICTION (“Prediction of Progression of Coronary Artery Disease and Clinical Outcome Using Vascular Profiling of Shear Stress and Wall Morphology”) Stone et al. [12]	506	Plaque burden ≥ 58%	PCI	1 year	17.57 (3.67–84.20)	<0.001
			ESS < 0.98 Pa			3.18 (1.20–8.43)	0.020
**NIRS**	AtheroRemo-NIRS Oemrawsingh et al. [59]	203	LCBI ≥ 43%	Non-culprit MACE	1 year	4.04 (1.33–12.29)	0.01
**IVUS & NIRS**	ATHEROREMO-NIRS and Integrated Biomarker Imaging Study 3 (IBIS-3) studies [46]	286	Max LCBI_4mm_ (per 100-unit increase)	Non-culprit MACE	4.1 years	1.22 (1.10–1.35)	<0.001
	Spectrum NIRS-IVUS registry [48]	202	MaxLCBI_4mm_ (per 100-unit increase)	Target vessel failure	3.5 years	1.6 (1.2–2.1)	0.0040
	LRP (Lipid Rich Plaque) Study [60]	1563	MaxLCBI_4mm_ (per 100-unit increase)	Non-culprit MACE	2 years	1.21 (1.09–1.35)	0.0004
**OCT, NIRS, IVUS & VH**	PREVENT (“The Preventive Coronary Intervention on Stenosis With Functionally Insignificant Vulnerable Plaque”, ClinicalTrials.gov Identifier: NCT02316886)	1600		Target vessel failure	2 years	Recruiting	
**NIRS & IVUS**	PROSPECT II (“Providing Regional Observations to Study Predictors of Events in the Coronary Tree”) [47]	898	High lipid content	Non-culprit MACE	3.7 years	OR 3.80 (1.87–7.70)	0.0002
			Plaque burden ≥ 70%			OR 5.37 (2.42–11.89)	<0.0001
			MaxLCBI_4mm_ ≥ 324.7 Plaque burden ≥ 70%			OR 11.33 (6.10–21.03)	

* All three epicardial coronary vessels (left anterior descending, left circumflex, right coronary) were analyzed in all of the studies included in the table. Abbreviations: CFD, computational fluid dynamics; ESS, endothelial shear stress; IVUS, intravascular ultrasound; LCBI, lipid core burden index; MACE, major adverse cardiovascular events; N, sample size; NA, not applicable; NIRS, near-infrared spectroscopy; OCT, optical coherence tomography; OR, odds ratio; PCI, percutaneous coronary intervention; VH, virtual histology.

### 3.3. Biomechanical Regulators of Atherothrombosis

**Figure 4:**
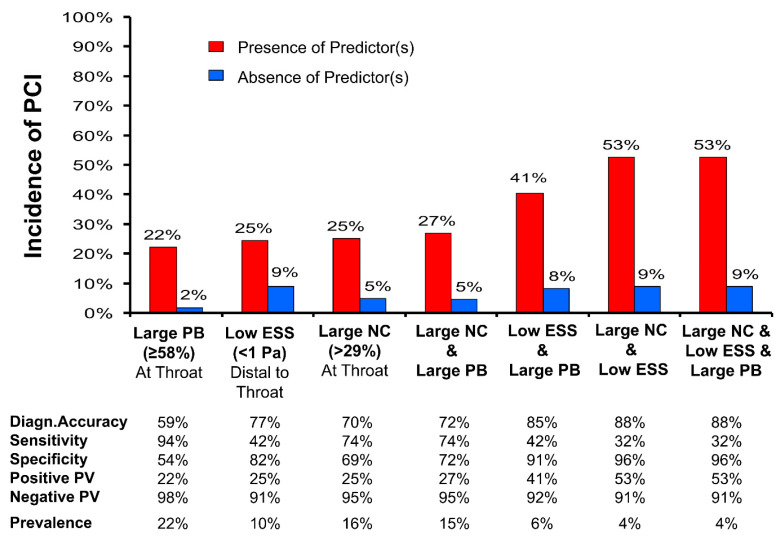
Prognostic performance of IVUS-based imaging (large plaque burden [PB] and large necrotic core [NC]) and hemodynamic (endothelial shear stress [ESS]) predictors of the occurrence of percutaneous coronary intervention (PCI) for baseline luminal obstructions due to symptoms or substantial lesion progression. The data on prevalence refer to one or more such baseline luminal obstructions per patient. PV: predictive value. Adapted from Papafaklis M, et al. (2016) [65], with permission from Elsevier.

Hemodynamic forces, primarily endothelial shear stress (ESS), have a pivotal role in cardiovascular pathophysiology and are inherently related to the focal nature of CAD. Low local ESS imposes a multifactorial effect on the arterial endothelium and is associated with the development and progression of atherosclerosis [61]. In vivo ESS assessment is accomplished by combining 3-D coronary imaging (e.g., coronary angiography with IVUS or OCT) with computational fluid dynamics (CFD) [62,63]. High-risk plaque features are typically associated with low ESS [64], while ESS is also an independent predictor of atherosclerosis progression and ACS [12]. The PREDICTION study showed that low ESS and plaque burden >70% can identify with a PPV of 41% which plaques are more likely to progress and should be treated with percutaneous coronary intervention (PCI). A PPV of 53% for identifying lesions prone to cause ACS has been shown when low ESS, a high plaque burden, and a large necrotic core are all present [65] (Figure 4), indicating that a combination of these predictors could improve prognostication of plaque vulnerability. Additionally, results from a post hoc analysis of the PROSPECT study have shown that low ESS at baseline provides substantial incremental value independent of aforementioned traditional factors in predicting MACE over a 3-year follow-up (54.9% for non-culprit lesions with baseline low focal ESS and high-risk anatomy vs. 19.5% for patients with non-culprit lesions with low ESS without high-risk anatomy; *p* = 0.004) [66,67].

Apart from ESS, blood flow also generates another hemodynamic force acting on plaques: the plaque structural stress (PSS). Circumferential and axial stresses resulting from blood pressure contribute to the total strain distribution within the vessel and are described as novel aspects of vulnerable plaques [68]. More specifically, circumferential tension stems from hydrostatic pressure, which applies an outward radial force on the arterial wall. The distribution of this pressure relies on the mechanical characteristics and the organization of the vessel components. Axial plaque stress (APS), on the contrary, derives from the longitudinal expansion of vessels because of the cyclical blood flow and motion of the heart. Analysis of these forces along the centerline isolates the longitudinal component of the hemodynamic stress. Obstructions of coronary flow can create pressure gradients across coronary plaques, causing an increase in axial tension and overall plaque stress, leading to plaque rupture. In a 3-D OCT study of patients presenting with ACS, culprit areas were exposed to higher APS at the time of the event. Although the number of study participants was small (only 15 patients), the multivariable analysis demonstrated that axial plaque stress was one of the most significant independent predictors of the location of the culprit lesions [69].

## 4. Challenges of the “Vulnerable Plaque” Concept

So far, published studies have failed to show significant clinical benefits for plaque imaging. Despite the independent association of image-based findings with clinical events, the highest positive predictive value achieved by intravascular imaging studies is only 53%. In patients with ACS, plaque rupture is often found far from baseline culprit lesions, suggesting that vulnerability may be dispersed in the coronary arteries [70]. This indicates that discerning a “vulnerable state” in a patient may be more important than identifying focal sites of vulnerability [14].

Moreover, the introduction of statin therapy with an improvement of the lipid profile, efforts to control tobacco abuse, and better management of insulin resistance/diabetes and hypertension have reshaped and stabilized plaques through an increase in the fibrous cap thickness, leading to a relative increase in superficial erosion compared to plaque ruptures regarding ACS [7,42,71,72]. In addition, an increase in non-ST segment elevation myocardial infarction (NSTEMI) has been observed [73]. These findings suggest a shift in the pathological mechanisms and presentations of ACS mainly due to primary prevention measures. Analysis of >1500 plaques showed that macrophage-rich (a classical component of ruptured plaques) atheromas have significantly reduced, proving the claim that the “vulnerable plaque” concept has receded in relevance [74].

“Vulnerable” characteristics of plaque morphology change over time with their susceptibility to rupture or erosion increasing or decreasing. Approximately 75% of TCFAs transform into thick-cap fibroatheromas or fibrotic atheromas within a year due to processes of rupture and healing, further supporting the concept of subclinical plaque alterations [75]. The final act of atherosclerosis, additionally, is regulated by numerous systemic factors such as blood viscosity, platelet activity, fibrinogen levels, and the interaction between the coagulation and fibrinolytic system, indicating once again the systemic nature of the disease.

A shift from the concept of the “vulnerable plaque” to the “burden of disease” through non-invasive imaging, combined with conventional risk factors and reflecting a “patient-centered” approach, is gaining ground lately [76,77,78,79,80].

## 5. Identifying the “Vulnerable Patient”

### 5.1. Risk Scores

A preventive approach or early prediction of CAD events in the asymptomatic population was the main notion that evolved in the era following the Framingham study. Risk scores have been developed to help physicians estimate the additive effect of different risk factors, discover high-risk individuals, and implement treatment approaches.

The Systematic COronary Risk Evaluation (SCORE) project is an easy-to-use stratification system focusing on the primary prevention of CVD in asymptomatic individuals without established CAD [81]. Risk factors constituting SCORE include sex, systolic blood pressure (SBP), dyslipidemia, smoking, and age. SCORE determines total cardiovascular risk rather than solely coronary heart disease risk. Furthermore, the SCORE project focuses only on fatal cardiovascular events rather than on the combined fatal and non-fatal events, as non-fatal events are dependent on definitions and methods used in their confirmation. Recently, the updated SCORE2 prediction model has been developed to estimate the 10-year risk of fatal as well as non-fatal CVD events in European individuals without previous CVD or diabetes aged 40–69 years [82]. Additionally, the SCORE2-Older Persons (SCORE2-OP) algorithm has also been derived to estimate the risk for the combined outcome of both fatal and non-fatal CVD events in adults aged 70 years or older in the next 5 and 10 years [83].

The “ARIC Coronary Heart Disease Risk Calculation” score for coronary events was based on the results of the Atherosclerosis Risk in Communities (ARIC) study [84]. Researchers assessed the prognostic value of several clinical variables. Low risk for events was associated with non-smokers, those having total cholesterol <200 mg/dL, high-density lipoprotein (HDL) > 60 mg/dL, SBP < 120 mm Hg, and not requiring antihypertensive treatment.

The “AtheroSclerotic Cardiovascular Disease (ASCVD) Risk Estimator” estimates the risk of ASCVD in the next 10 years. The estimator was developed using data from large, diverse cohorts, including the ARIC [84], Cardiovascular Health [85], and the Coronary Artery Risk Development in Young Adults (CARDIA) [86], as well as with the Framingham Original and Offspring Study cohorts [87]. The ASCVD estimator accompanied the “2013 ACC/AHA Guideline on the Assessment of Cardiovascular Risk” [88,89] and the “2013 ACC/AHA Guideline on the Treatment of Blood Cholesterol to Reduce Atherosclerotic Cardiovascular Risk in Adults” [90]. The statistically significant variables included in the equations are age, total cholesterol, HDL, SBP (with treatment status), diabetes, and smoking. High risk is defined as ≥7.5%.

### 5.2. Biomarkers, Antibodies, and Genetics

Serological biomarkers have long been studied as potential predictors of CAD. Inflammation has a key role in atherosclerotic disease [91] and it therefore comes as no surprise that circulating levels of inflammatory markers including cytokines, such as interleukin-1β and interleukin-6, have been shown to increase in atherosclerosis [92,93,94,95]. Additionally, various studies have shown that adhesion molecules that mediate the recruitment of leukocytes at sites of inflammation, such as endothelial-leukocyte adhesion molecule-1 (E-selectin) and intercellular adhesion molecule-1 (ICAM-1), are increased in individuals with atherosclerosis [96,97]. Among females in the Nurses’ Health Study and males participating in the Health Professionals Follow-study, increased inflammatory biomarkers, particularly C-reactive protein (CRP), indicated a higher risk of CAD [98]. Recently, a systematic review of meta-analyses once again indicated that CRP can be a marker with strong predictive potential along with fibrinogen, apolipoprotein (Apo) B, HDL, and Vitamin D [99].

Numerous other biomarkers have also been shown to increase in atherosclerosis. Elevated homocysteine levels (>12 μmol/L) have been shown to predict the progression of coronary plaque burden [100]. Furthermore, plasma amyloid-β (1–40) (Aβ40) has been associated with the presence of subclinical CAD in individuals without clinically overt CAD [101,102], arterial stiffness progression in young healthy individuals, as well as with cardiovascular mortality and MACE in patients with CAD [103]. Baseline circulating levels of Aβ40, as well as circulating cathepsin S levels, can predict mortality and improve risk stratification of patients with NSTEMI after adjusting for the Global Registry of Acute Coronary Events (GRACE) score [104,105]. Cathepsin S levels have also been associated with vascular aging, arterial stiffening, and atherosclerotic disease development [106,107].

Fibroblast growth factors (FGFs) have been considered potential targets for the prevention and treatment of cardiovascular events [108]. Patients with CAD have also been found to have lower levels of FGF19 than those without CAD, adjusting for other factors, while FGF19 was also an independent predictor of the extent of atherosclerosis. FGF23 is associated with CAD risk factors such as apolipoprotein A1 and HDL in subjects [109]. In stable CAD, increased levels of FGF23 have also been associated with cardiovascular mortality and heart failure [110]. FGF21 levels are strongly related to traditional CVD risk factors such as dyslipidemia, hypertension, diabetes, and obesity [111,112]. FGF21 is an important regulator in several metabolic pathways including glucose and lipid metabolism, suggesting a potentially protective effect, contributing to cardiovascular risk reduction [113,114].

The immune system can play pathological or protective roles in atherosclerosis [115,116]. Even though circulating immunoglobulins are not typically considered relevant to clinical cardiovascular disease, there is significant evidence suggesting links with atherosclerosis. A nested case-control study of the Anglo-Scandinavian Cardiac Outcomes Trial suggests that total serum IgG levels are strongly and independently associated with reduced risk of cardiovascular events in individuals with hypertension and improve prediction beyond traditional risk predictors, such as CRP [117]. Serum IgM level is also associated with reduced risk of CAD events, but to a lesser extent than IgG.

Antinuclear antibodies (ANAs) are another independently associated predictor of all-cause mortality, cardiovascular death, and ASCVD (cardiovascular death, myocardial infarction, coronary revascularization, and stroke), as was shown in a representative, multiethnic cohort [118]. ANAs, typically found in individuals with autoimmune disease, can detect individuals at increased risk of death and ASCVD, independently of traditional risk factors or the presence of autoimmune disease.

The genetic profile, the principal substrate of conventional risk factors and disease, emerges as an additive tool in the quest to identify the “vulnerable patient”. Results from numerous prospective and retrospective studies have shown that genetic risk scores (GRSs), derived from the identification of single nucleotide polymorphisms (SNPs) related to CAD, can be associated with adverse cardiovascular events. Numerous SNPs have been identified as predictors of CAD, independent of self-reported family history (with the heritability of CAD being well documented), and can thus be especially useful in young individuals’ profile characterization [119,120].

## 6. Non-Invasive Imaging

### 6.1. Indications

Even though invasive diagnostic tests are invaluable in identifying significant CAD and prognosticating disease progression, they impose an intrinsic risk to patients due to the invasive nature of coronary catheterization. For this reason, there is a clear clinical need for non-invasive techniques that can provide insight into distinguishing “vulnerable plaques” and prognostic stratification, particularly in asymptomatic individuals wherein the use of invasive techniques is not suitable. To date, the most established non-invasive diagnostic modalities include exercise stress testing, coronary calcium scoring, CCTA, magnetic resonance coronary angiography, and positron emission tomography. Although exercise stress testing is a low-cost, low-risk diagnostic method that has been validated, it has limited sensitivity and specificity, and cannot identify the extent and location of CAD [121]. As a result, although it is recommended for the evaluation of chest pain in intermediate-risk patients, it is becoming increasingly replaced by other non-invasive imaging modalities, such as CCTA.

In cases when ischemia is clinically suspected, non-invasive imaging modalities can improve the detection of an obstructive plaque causing significant myocardial blood flow compromise. Additionally, in asymptomatic individuals, non-invasive imaging can help identify the risk of cardiac events. Non-invasive imaging modalities lie in the “grey zone” between the plaque and the patient-centered concept, providing a less interventional approach than IVUS or OCT technologies in the pursuit to find the “vulnerable plaque” and a more advanced approach in the identification of the “burden” of disease than risk scores and circulating biomarkers.

### 6.2. Subclinical Atherosclerosis

The BioImage Study aims to explore the associations between imaging analysis and levels of biomarkers along with their capability to predict ACS in asymptomatic individuals [122]. The diagnosis of subclinical atherosclerosis was established with one or more of the following: (i) the presence of carotid plaque; (ii) abnormal carotid intima-media thickness (IMT) (exceeding age-stratified cutoff values); (iii) abnormal coronary artery calcification score (CACS) (Agatston score) defined as: “a value above the 75th percentile adjusted for age and gender”; (iv) the presence of abdominal aortic aneurysm; and (v) abnormal ankle-brachial index (ABI) (<0.9). This noninvasive screening can significantly ameliorate the cardiovascular risk classification of the general population.

### 6.3. Computed Tomography and Positron Emission Tomography

**CACS** refers to the assessment of coronary artery calcification performed on non-contrast computed tomography scans of the heart. Calcium is defined as a lesion of more than 1 mm^2^ with a density >180 Hounsfield units, and it is calculated using the Agatston score, which takes into account both calcium density and distribution. In asymptomatic low-to-moderate risk individuals, CACS has a good negative predictive value, but a low positive predictive value, and, thus, additional imaging tests are often required. In patients with suspected angina, CACS has an increased rate of false-negative results, which is unsurprising since it is well-established that non-calcified plaques can also rupture, resulting in MI. Even though numerous plaques do not contain calcium that can be detected in CT, the total CACS of an individual can provide acceptable information for coronary obstruction and overall plaque burden, and it thus provides an incremental predictive value when added along with other clinical risk factors and biomarkers in well-established risk estimators, including the Framingham score. This has been confirmed in large trials, including the Multi-Ethnic Study of Atherosclerosis (MESA) trial [123,124].

**Figure 5:**
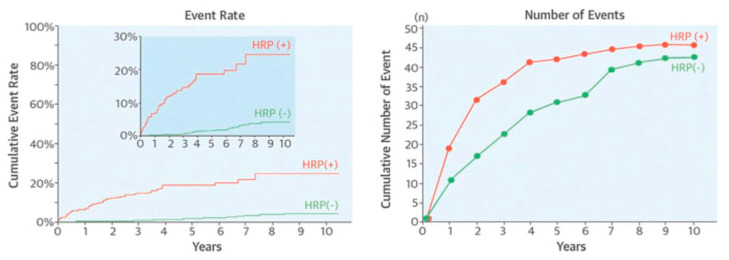
Prediction of acute coronary syndromes using computed tomography angiography. High-risk plaque (HRP) using CTA is defined based on low attenuation (≤30 Hounsfield units) and/or positive remodeling. In this study of 3158 subjects, 294 (9.3%) had HRP(+) and 2864 (90.7%) did not have HRP [HRP(−)]. During follow-up (3.9 ± 2.4 years), 48 (16.3%) of the subjects with HRP and 40 (1.4%) of the subjects without HRP developed acute events. Therefore, the event rate (**left**) was substantially higher for HRP(+) compared to HRP(−) (green), but the total number of events in the two groups was essentially the same (**right**). Additionally, serial CTA imaging showed plaque progression from HRP(−) to HRP(+) leading to acute coronary events in some cases. While the identification of HRP is important, these observations highlight the diffuse process and complex evolution of atherosclerosis, which is responsible for acute events. Adapted from Motoyama S. et al. (2015) [127], with permission from Elsevier.

**Coronary computed tomography angiography (CCTA)** is considered to be a first-line diagnostic technique in patients with suspected CAD [125]. CCTA can detect luminal stenoses and can improve clinical outcomes by improving the targeting of symptomatic and preventative therapies. Additionally, coronary CTA can recognize high-risk plaque characteristics associated with an increased risk of adverse events [126]. Characterization of coronary geometry and plaque composition, quantification of plaque burden, as well as 3-D vessel reconstruction for blood flow simulation and estimation of hemodynamic forces including ESS, is also feasible using CCTA. Identification of a high-risk plaque based on CT findings, such as positive remodeling and low attenuation regions, can independently predict the development of ACS [127] (Figure 5). Furthermore, a subgroup analysis showed that CT-derived low ESS was associated with an increase in plaque burden and a decrease in lumen area at follow-up [128,129], confirming the results of previous invasive imaging-based studies [12].

More recent studies have focused on detecting coronary inflammation, a key mediator of atherosclerosis. Inflammation-induced changes that can present even before the development of plaques can be quantified as perivascular attenuation gradients estimating the CCTA-derived Fat Attenuation Index (FAI) [129]. The CRISP-CT (“Cardiovascular Risk Prediction using Computed Tomography”) study showed that perivascular FAI has incremental prognostic value beyond traditional risk factors [130]. Moreover, there is a remarkable improvement in risk estimation for cardiac mortality and other causes of death with the addition of FAI in CCTA interpretation. Therefore, perivascular fat could set the scene for personalized risk assessment in primary and secondary prevention.

A combination of **positron emission tomography (PET) and CCTA** has been recently introduced as a propitious non-invasive technology that combines functional imaging with anatomical data [131,132]. In stable CAD, the radioactive tracer ^18^F-sodium fluoride uptake, suggesting inflammation and macrophage accumulation, can help discriminate coronary plaques with high-risk features identified using IVUS [133]. Consequently, PET-CT methodology seems to help recognize active lesions that could ultimately result in adverse cardiovascular events.

## 7. Challenges of the “Vulnerable Patient” Concept

Contrary to invasive imaging technologies that focus on patients diagnosed with CAD, non-invasive strategies provide opportunities for identifying coronary atherosclerosis in asymptomatic people [134]. In asymptomatic individuals, the event rate is significantly lower, and, thus, regardless of whether future studies prove the prognostic importance of imaging, the cost-effectiveness of such methods must be analyzed before incorporating them in clinical practice [3]. The stepwise approach used in the BioImage study could be an option. Assessment of risk using clinical scores and measurements of biomarkers can be used at first to identify patients as intermediate/high-risk. Non-invasive imaging will be offered to these individuals to decide if an ACS is likely and, thus, they need to receive more aggressive treatment [3]. Meanwhile, the Danish Cardiovascular Screening (DANCAVAS) trial, which is a population-based, randomized, controlled trial involving 46,611 males aged 65 to 74 years, showed that comprehensive screening for cardiovascular disease did not manage to significantly reduce the risk of death from any cause in the first 5 years of the study [135].

## 8. Future Implications

A combination of invasive and non-invasive imaging modalities with modeling of patient characteristics is likely to provide a more precise identification of “vulnerable plaques” and “vulnerable patients” in the near future, promising a more tailored management personalized to each patient. Evidence suggests that, in patients with suspected CAD, non-invasive imaging, serum analysis for biomarkers, and genetic assessment could each separately help to identify the presence of CAD, and thus their combination could potentially enhance the performance of risk stratification for future ACS. Additionally, patients undergoing invasive coronary angiography due to ACS or due to the presence of findings from non-invasive modalities that require further invasive interrogation may also benefit from extensive imaging with intravascular diagnostic techniques and hemodynamic profiling (e.g., ESS) of non-culprit lesions along with assessment of biomarkers and genetics. This could provide an incremental value to risk assessment of future events arising from mild or moderate lesions. Before such strategies are implemented in clinical practice, further research investigating their clinical benefit as well as their cost-effectiveness is needed. Future studies in well-selected target groups (e.g., patients with suspected CAD and patients with known CAD) should evaluate the benefit of multilevel assessment to identify “vulnerable plaques and patients” at risk for future cardiovascular events.

## 9. Conclusions

Despite striking developments in diagnosis and treatment, CAD is a principal cause of mortality and morbidity globally. Finding individuals at risk as well as plaques prone to lead to cardiovascular events is important for improving prognostication and optimizing treatment. The “vulnerable plaque” concept has proved invaluable in directing research efforts and gaining knowledge of the underlying pathophysiology of ACS. Invasive imaging tools (such as IVUS, OCT, and NIRS), and hemodynamic profiling provide unique insights into plaque anatomy, and allow in vivo identification of TCFAs and other high-risk plaque elements, such as lipid component, neovessels, plaque erosion, macrophage accumulation, and low ESS, and have boosted our knowledge about coronary events. However, this invasive-only approach towards ACS has failed to demonstrate high prognostic value, and its clinical utility is called into question. Risk scores, identification of subclinical atherosclerosis, biomarkers, antibodies, and genetic predisposition accompanied by non-invasive imaging (MSCT, PET-CT) also hold the genuine promise of a safer, more time-efficient and clinically broader (asymptomatic population included) approach to CAD. As the debate is ongoing, the various diagnostic methods seem to complement each other, dealing with different aspects of the disease, characterizing the “vulnerable plaque” occurring in a “vulnerable patient”, and contributing to a holistic understanding of the fundamental causes of acute coronary events.

## Data Availability

Not applicable.
